# Iron deposition in multiple sclerosis: overall load or distribution alteration?

**DOI:** 10.1186/s41747-022-00279-9

**Published:** 2022-09-08

**Authors:** Eman Hamdy, Aya Abdel Galeel, Ismail Ramadan, Dina Gaber, Haytham Mustafa, Jaidaa Mekky

**Affiliations:** 1 Department of Neurology, Faculty of Medicine, Alexandria University, Alexandria, Egypt; 2 Department of Radiology, Faculty of Medicine, Alexandria University, Alexandria, Egypt; 3 Philips Healthcare, Cairo, Egypt

**Keywords:** Brain, Iron, Image processing (computer-assisted), Magnetic resonance imaging, Multiple sclerosis

## Abstract

**Background:**

Though abnormal iron deposition has been reported in specific brain regions in multiple sclerosis (MS), no data exist about whether the overall quantity of iron in the brain is altered or not. We aimed to determine whether the noted aberrant iron deposition in MS brains was a problem of overall load or regional distribution in a cohort of MS patients.

**Methods:**

An experienced neuroradiologist, a radiology software engineer, and four neurologists analysed data from quantitative susceptibility maps reconstructed from 3-T magnetic resonance brain images of 30 MS patients and 15 age- and sex-matched healthy controls. Global brain iron load was calculated, and the regional iron concentrations were assessed in 1,000 regions of interest placed in MS lesions in different locations, normal appearing white matter, thalami, and basal ganglia.

**Results:**

Global brain iron load was comparable between patients and controls after adjustment for volume (*p* = 0.660), whereas the regional iron concentrations were significantly different in patients than in control (*p* ≤ 0.031). There was no significant correlation between global iron load and clinical parameters, whereas regional iron concentrations correlated with patients’ age, disease duration, and disability grade (*p* ≤ 0.039).

**Conclusions:**

The aberrant iron deposition noted in MS seems to be a problem of regional distribution rather than an altered global brain iron load.

**Supplementary Information:**

The online version contains supplementary material available at 10.1186/s41747-022-00279-9.

## Key points

- Global brain iron load in multiple sclerosis (MS) is comparable to controls.
- Iron aberrant distribution in MS is a problem of regional distribution, not of global load.
- Regional, but not global, brain iron load is correlated with clinical characteristics.

## Background

Iron has been consistently noted to be aberrantly deposited in the brains of multiple sclerosis (MS) patients even after adjustment for the patients’ age and brain atrophy [1, 2]. Iron was found to be elevated in deep grey matter, *i.e*., caudate and putamen [3], and reduced in thalami [4–6] and normal-appearing white matter (NAWM) [7–9] in patients with MS when compared to their healthy counterparts [2, 10–12]. It was also found to be elevated around the MS plaques [13] and reduced inside the plaques [14, 15]. The data, however, are conflicting among the studies. Iron aberrant deposition in MS was reported to occur in almost all stages and phenotypes of MS, starting early at the clinically isolated syndrome phase [11]. This suggested that iron has a contributing role in the pathogenesis of the disease even in early stages, not a mere consequence of myelin damage and cell destruction [16].

Being a consistent finding in different studies, the research was directed towards identifying its correlation with the clinical profile [9, 17, 18]. Though the results are conflicting between the studies, the aberrant iron deposition in specific brain regions was reported to be mainly related to the patients' age, disease duration, and degree of disability assessed by the expanded disability status scale (EDSS) [19, 20]. Iron deposition around the edges of MS lesions was reported to be a marker of poor disability progression [21].

The exact mechanism of iron involvement in the pathogenesis of MS remains elusive. To date, it is not obvious whether iron deposition in MS patients is just an epiphenomenon, a consequence of the ongoing pathology, or an actual mediator of disease pathogenesis [22]. Initial attempts to understand the etiopathogenesis of iron deposition in MS proposed a theory of systemic iron overload [23, 24]. However, peripheral markers of iron metabolism and hemochromatosis genes were not different in MS patients compared to controls [25–27]. These findings negated the systemic iron overload problem in MS; and concluded a focal iron pathology confined to the central nervous system (CNS) [28]. Several postulations were made to explain the noted aberrant iron deposition in the CNS of MS patients. Altered iron influx or clearance from the brain via a disrupted blood-brain barrier (BBB) was proposed [28, 29]. Chronic cerebrospinal venous insufficiency, venous congestion, and subsequent red blood cell extravasation and iron deposition were also suggested [30]. Accordingly, several trials were conducted to evaluate the efficacy of chelation therapy and endovascular interventions in MS [31, 32]. The results of these trials, however, were essentially negative [31, 32]. Recent data suggested that the increased iron concentration in specific regions in MS brains is partially explained by volume loss without concomitant loss of iron load [33]. This, however, does not explain the observed low iron concentration in other regions such as the thalami.

While the literature points to a focal CNS iron pathology, it remains unclear whether the overall CNS iron load in MS patients is increased, reduced, or altered. Clarification of this point would be of help for future therapeutic implications. The chelation therapeutic agents used in the available studies in the literature do not cross the BBB [31]. Whether there is a need to develop chelation agents that can cross the BBB or not depends on the overall brain iron load. The currently published studies reported abnormal iron quantities in specific brain areas, but none mentioned if the global brain iron load was altered.

Therefore, in this study, we aimed to assess the global iron load as well as the regional iron concentrations in different locations (MS lesions, NAWM, thalami, and basal ganglia) in a cohort of MS patients in comparison to healthy controls and to assess their correlation with the clinical profile.

## Methods

### Study design and patient selection

This was an observational cross-sectional study conducted on 30 adult patients with relapsing-remitting MS (RRMS) diagnosed according to the revised 2017 McDonald’s criteria [34] and 15 age- and sex-matched healthy controls. Of the 30 patients recruited, 10 had benign MS (EDSS ≤ 2 after the first 5 years of disease onset [35]) and were on interferon-beta therapy, 10 had aggressive MS (EDSS ≥ 4 within the first 5 years of disease [36]) and were on interferon-beta therapy, and 10 patients were disease-modifying therapy (DMT)-naïve. All recruited patients had a disease duration between 5 to 10 years to limit the potential confounding effect of disease duration on brain iron concentrations [37].

### Imaging processing and iron measurement

All patients and control subjects underwent 3-T magnetic resonance imaging (Philips Ingenia 3-T MRI-scanner, Philips Healthcare, the Netherlands) using a 32-channel head coil. The protocol included three-dimensional (3D) T1-weighted, two-dimensional T2-weighted, 3D fluid-attenuated inversion recovery (FLAIR), regular diffusion-weighted images echo-planar imaging, and multi-echo susceptibility-weighted images (SWI) obtained with thin cuts at 0.5 mm. The obtained multi-echo SWI of all subjects was processed as previously described by Meineke et al. [38] to reconstruct quantitative susceptibility maps (QSM) (field of view, anteroposterior, feet-to-head, right-to-left 240 × 145 × 210 mm^3^; acquired voxel 0.6 × 0.6 × 2.0 mm^3^; flip angle 14°; echo time 3.5 ms; Δ echo time 4 ms; 7 echoes; repetition time 31 ms; bipolar readout; bandwidth 275.9 Hz/vx; sensitivity encoding, Phase/Slice 1.8 × 1.2) and a T1-weighted magnetisation-prepared turbo field-echo sequence, used for model-based segmentation (field of view 240 × 240 × 170 mm^3^; acquired voxel 0.94 × 0.94 × 1.0 mm^3^; flip angle 8°, echo time 8 ms; turbo factor 222; inversion delay 1,000 ms; bandwidth 191.5 Hz/vx; sensitivity encoding 1.0 × 2.2).

**Fig. 1:**
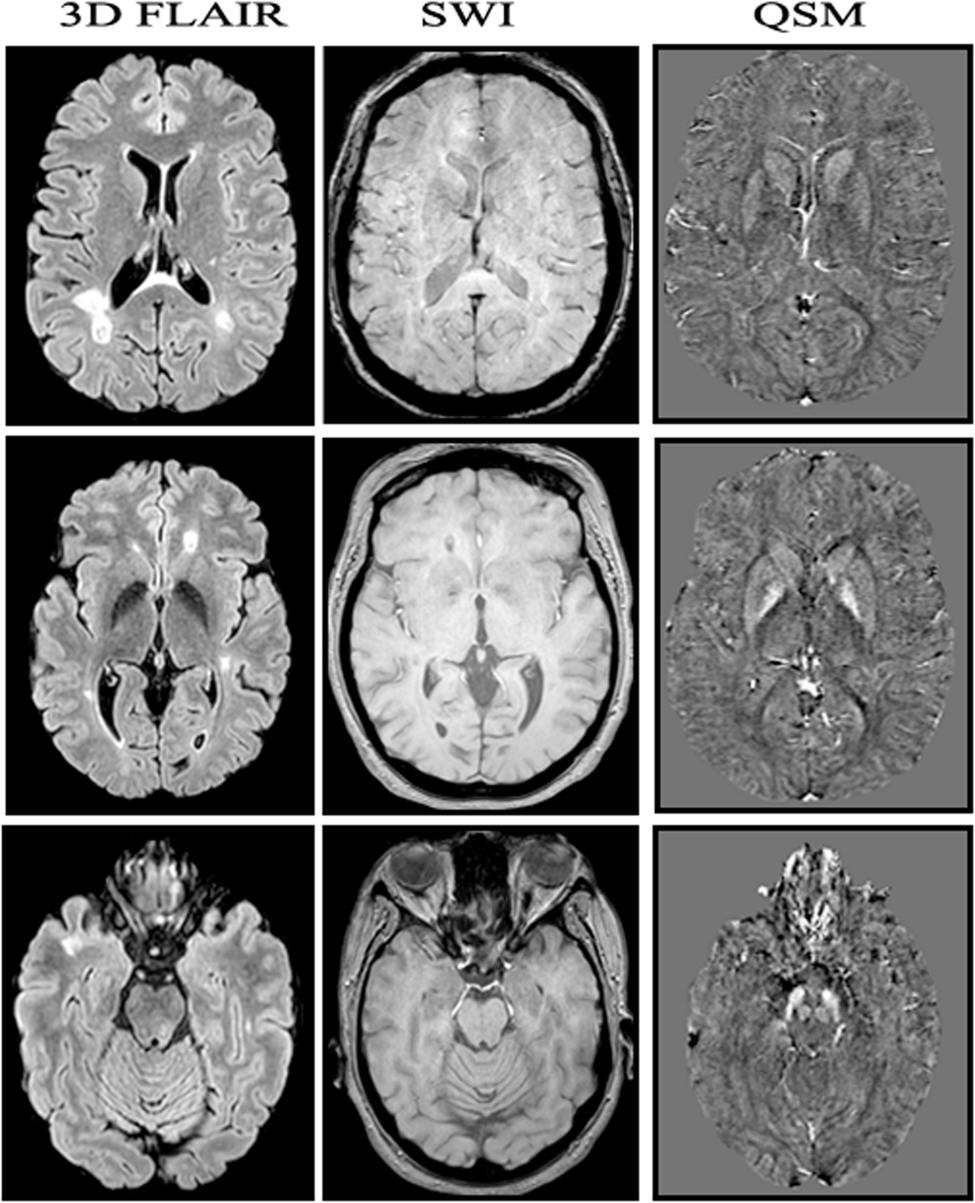
Magnetic resonance image of the brain of a 36-year-old lady diagnosed with relapsing-remitting multiple sclerosis. Three different imaging series of corresponding axial cuts are demonstrated, *i.e*., three-dimensional fluid-attenuation inversion recovery images, susceptibility-weighted images, and the reconstructed quantitative susceptibility maps

Assuming there was only one volume in the DICOM (Digital Imaging and COmmunications in Medicine) directory in SWI, echo was loaded at TEs of 7.2 ms, 13.4 ms, 19.6 ms, and then 25.8 ms. A mask was generated from the first echo of the QSM scan using some magnitude thresholding and then, the skull-stripping was started. After that, field mapping from echoes was performed, rescaled from KHz to Hz, and the weight data were calculated. Final QSM maps were generated (Fig. 1) where iron quantification was accessible. For iron quantification, Multi-image Analysis Graphical User Interface (Mango) software version 4.0.1 (University of Texas, San Antonio, USA) for windows was used [39–42].

**Fig. 2:**
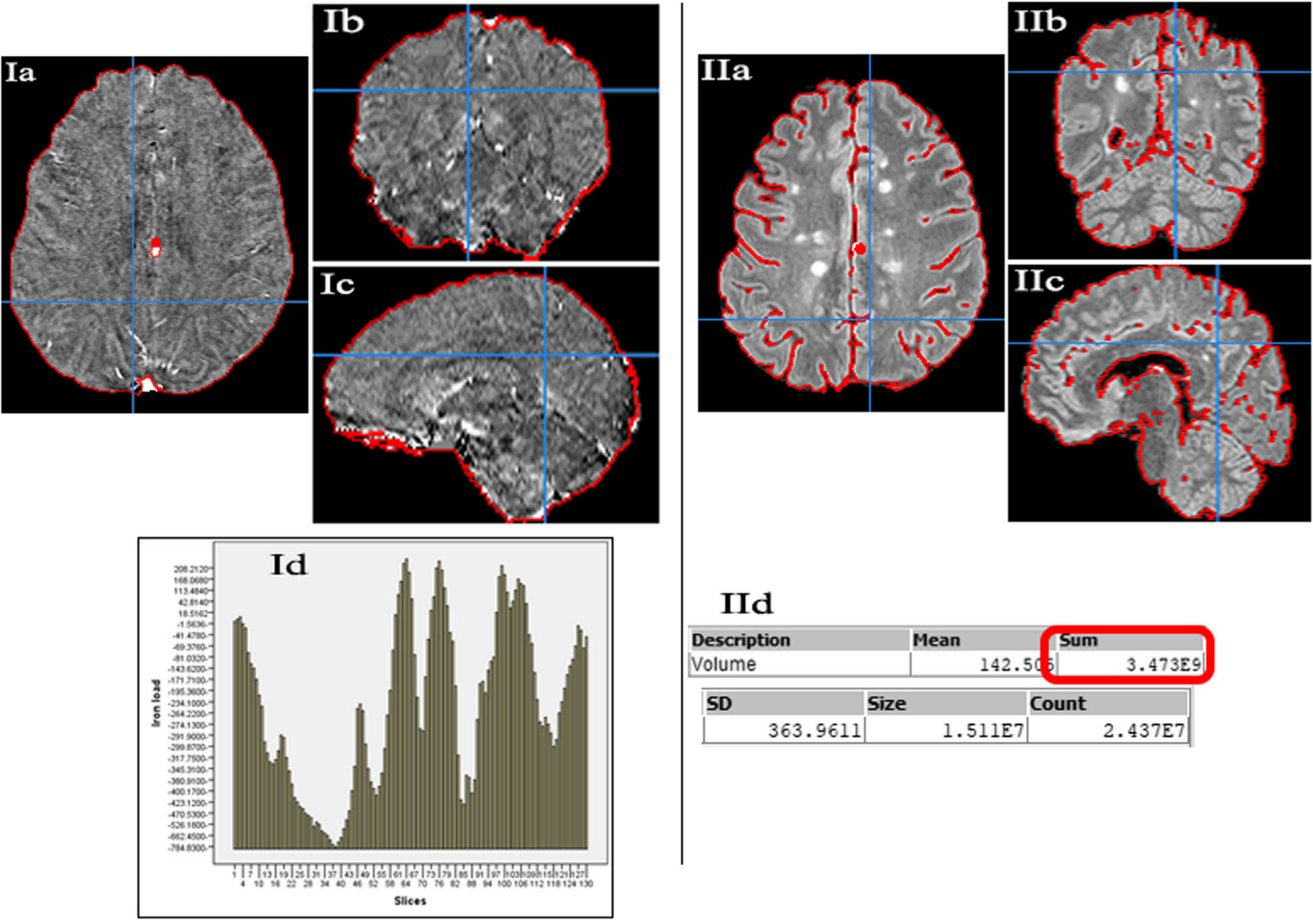
Measurement of global iron load (**I**) and brain volume (**II**). **Ia**, **Ib**, and **Ic** show the brain selected in quantitative susceptibility maps in axial, coronal, and sagittal views, respectively. Image Id shows the iron load (vertical axis) in 130 slices (horizontal axis) of one subject. The global iron load for each subject was the sum of the iron concentration in the 130 slices. Images **IIa**, **IIb**, and **IIc** show the brain selected in three-dimensional fluid-attenuation inversion recovery series to measure the brain volume. The results of volume analysis for each subject are the mean, sum, standard deviation, size, and count as shown in **IId**. The sum was the volume of the brain included in the final analysis

To measure global iron load, the brain was first extracted and selected using a threshold to a region of interest (ROI) as demonstrated in the software manual [39]. The sum iron load of all slices (130 slices per subject) was calculated (Fig. 2I). Brain volume for all subjects was also measured to adjust for the potential impact of brain atrophy in MS patients on the calculated iron load. The brain volume was measured from fluid-attenuated inversion recovery images (Fig. 2II). The brain was selected, excluding the ventricular system and the sulci; then, the volume was measured as demonstrated in the Mango software manual [39].

**Fig. 3:**
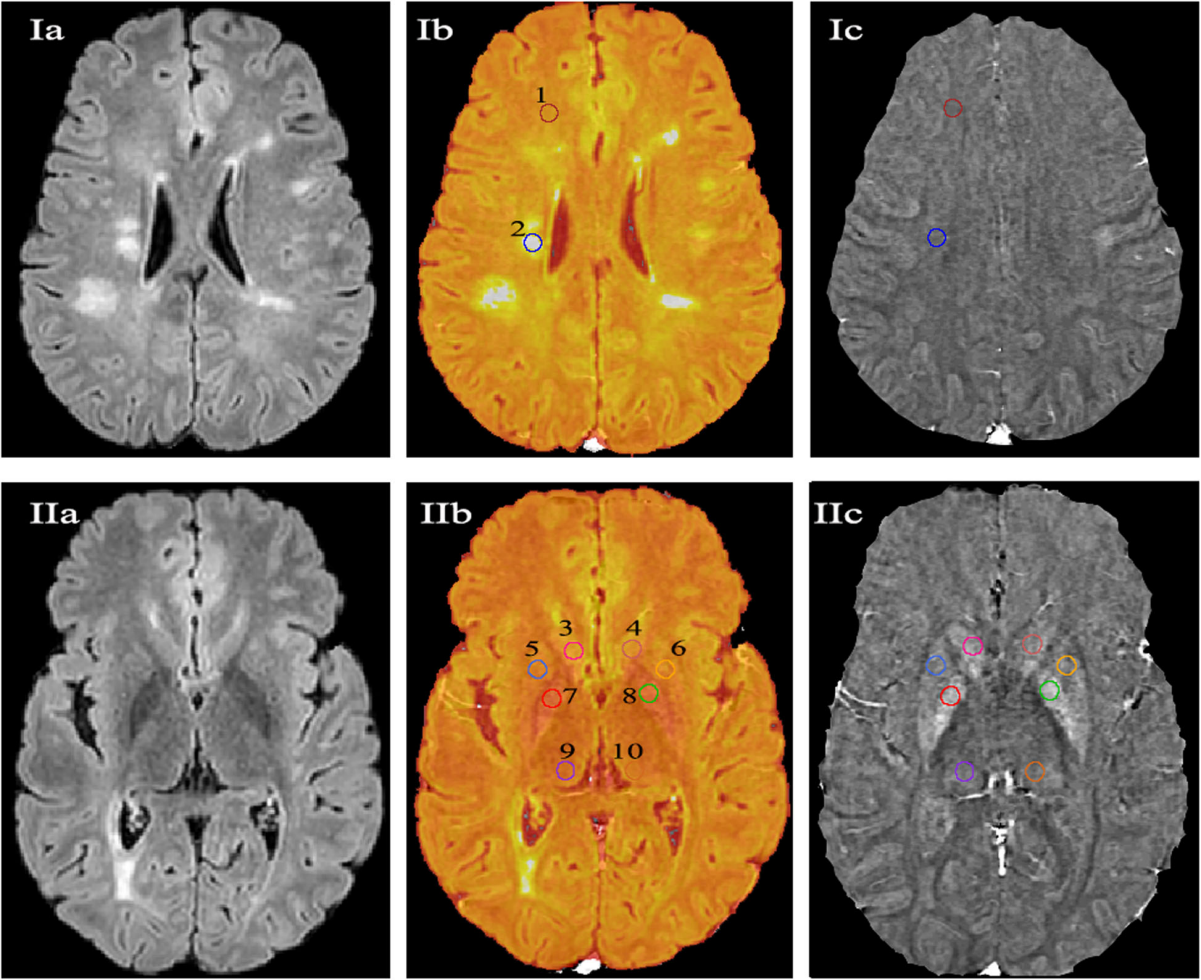
Measurement of regional iron in a 25-year-old man with MS. Images **Ia**, **Ib**, and **Ic** represent the steps of measuring iron concentrations in the normal appearing white matter (region of interest [ROI] 1) and multiple sclerosis lesions (ROI 2). **Ia** is an axial three-dimensional fluid-attenuation inversion recovery (3D FLAIR) film demonstrating the MS lesions. **Ib** is the 3D FLAIR image overlayed onto the quantitative susceptibility mapping, and **Ic** is the quantitative susceptibility map. Similar steps are demonstrated in **IIa**, **IIb**, and **IIc** to measure iron concentrations in the deep grey matter: caudate (ROIs 3 and 4), putamen (ROIs 5 and 6), globus pallidus (ROIs 7 and 8), and thalami (ROIs 9 and 10).

**Fig. 4:**
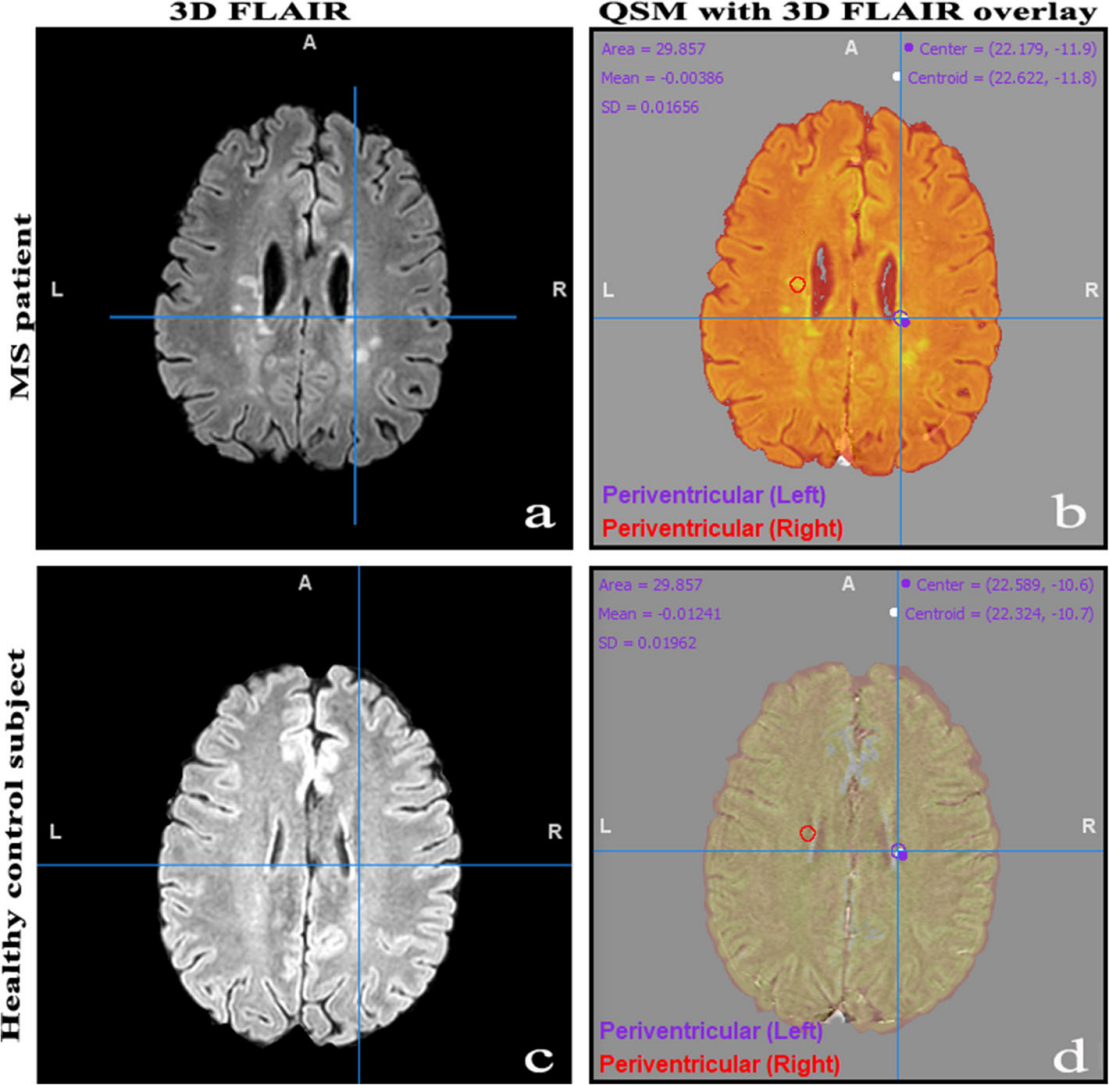
Measuring regional iron load in a 39-year-old man with relapsing-remitting multiple sclerosis (**A**, **B**) in periventricular lesions and measuring iron load in corresponding locations in a healthy 44-year-old male control subject. **A** represents an axial three-dimensional fluid-attenuation inversion recovery (3D FLAIR) film of the patient demonstrating two periventricular lesions (right and left). The 3D FLAIR film was overlayed on the quantitative susceptibility map as depicted in image **B** and the transparency was set to 50% to visualise both the quantitative susceptibility mapping (QSM) and the 3D FLAIR images. Two regions of interest (ROIs), shown as red and violet circles, were placed in the periventricular lesions and the ROIs were analysed on the QSM image. **C** Represents a corresponding axial 3D FLAIR cut of a healthy man. Similar steps were performed to measure iron in approximate locations corresponding to the MS periventricular lesions

To measure regional iron concentrations, fixed size (3-mm spheres) ROIs were placed in MS lesions at different locations (periventricular, cortical/juxtacortical, and infratentorial), NAWM, thalami, and basal ganglia (caudate, putamen, and globus pallidus) (Fig. 3). Because MS lesions were not visible on quantitative susceptibility maps (Fig. 3(Ic)), a 3D FLAIR image (Fig. 3(Ia)) was overlaid over the QSM image to place the ROIs inside the MS lesions (Fig. 3(Ib). The overlay was a semiautomatic image coregistration tool in the Mango software. Before further analysis, the radiologist manually revised that the images are strictly coregistered for all aspects, including the cortical and the ventricular outlines. The overlay was removed, and the iron was quantified in the QSM image (Fig. 3-Ic). In the healthy controls, ROIs were placed in similar locations (Fig. 4).

### Data collection and statistical analysis

Along with the radiological data, we collected demographic and clinical data from the patients’ medical records, and assessment of the EDSS, nine-hole peg test, timed-25-foot walking (T25FW) test, and symbol digit modality test were performed during an interview with the patient. Data were analysed using IBM SPSS software package version 20.0 (IBM Corp., Armonk, NY, USA). Kolmogorov-Smirnov test was used to verify the normality of the distribution of variables. Mean and standard deviation (SD) were used to summarise parametric continuous variables. Median and interquartile ranges were used to summarise nonparametric continuous variables. Categorical variables were summarised as numbers and percentages. To compare two groups, Student’s *t* and Mann-Whitney *U* tests were used for parametric and nonparametric variables, respectively. Kruskal-Wallis test was used to compare nonparametric variables between more than two groups. Spearman coefficient was used to test the correlation between nonparametric continuous variables. The significance of the results was judged at 0.050.

### Ethical considerations

Ethical approval was obtained from the ethical committee of Alexandria University Faculty of Medicine (Institutional review board protocol number: 00012098), which operates according to the International Conference of Harmonization Good Clinical Practice and applicable local and institutional regulations and guidelines [35]. The ethical committee has a federal-wide assurance [36] from 2010 (number: 00018699). The EC approved this study on the 24th of October 2019 (serial number: 0201291). Written informed consent was obtained from all subjects prior to recruitment to the study.

## Results

Sample demographic and clinical characteristics are summarised in Table 1. Age, sex, and disease duration were comparable between the studied groups (*p* ≥ 0.075). In Supplementary Table 1, the number of ROIs placed at each location is detailed. Table 2 summarises the global and regional iron load in the patients and healthy controls. Even after adjusting the brain volume, patients with MS had a similar global iron load to the healthy controls. The median global iron load was -20.994 parts per billion (ppb)/cm^3^ in MS patients and -14.577 ppb/cm^3^ in controls (*p* = 0.660). No significant differences were seen within the subgroups of patients.

**Table 1:** Sample descriptive analysis (*n* = 45)

	MS patients (*n* = 30)				HC (*n* = 15)	*P*_1_	*P*_2_
	Total	Benign (*n* = 10)	Aggressive (*n* = 10)	Naïve (*n* = 10)			
**Age in years**^**a**^	34.5 ± 7.51	30.2 ± 5.63	37.3 ± .74	36.3 ± 10.09	36.27 ± 10.27	0.512	0.075
**Gender,** ***n*** **(%)**							
– Male	7 (23.3)	2 (20)	2 (20)	3 (30)	4 (26.7)	0.540	0.830
– Female	23 (76.7)	8 (80)	8 (80)	7 (70)	11 (73.3)		
**Disease duration**^**b**^	7.0 (6–7.25)	6 (5–7.25)	6.50 (5.75–7.25)	7 (6–9)	–	–	0.379
**DMT duration**^**b**^	2 (0–3)	2.5 (1.75–3.5)	3 (2.75–4.25)	–			< 0.001*
**EDSS**^**b**^	3.5 (2.5–4.13)	2 (2–2)	4 (4–5.87)	3.5 (2.4–6.0)	–	–	0.001*
**Total number of relapses**^**b**^	5.0 (4.0–7.0)	4 (3.75–6.25)	7 (6–11.25)	4.5 (2.75–6)	–	–	0.041*
**SDMT**^**a**^	33.30 ± 14.99	39.8 ± 13.06	27.1 ± 12.21	3.0 ± 17.76	–	–	0.168
**9-HPT**^**b**^							
– Right hand	23.86 (22.03–31.45)	21.6 (19.4–23.2)	28.1 (23.8–43.1)	24.1 (22.2–34.1)	–	–	0.011*
– Left hand	28.11 (24.52–37.63)	25.1 (23.0–27.3)	30.9 (26.3–46.6)	30.8 (26.5–39.1)	–		0.052
**T25FW test**^**b**^	8.84 (7.09-12.82)	6.9 (6.5–8.3)	12.5 (8.6–17.4)	11.1 (7.4–16.6)	–	–	0.009*

*9-HPT* Nine hole-peg hole test, *DMT* Disease-modifying therapy, *EDSS* Expanded disability status scale, *HC* Healthy controls, *MS* Multiple sclerosis, *n* Number, *SDMT* Symbol digit modality test, *T25FW* Timed 25-foot walking, *p*_*1*_^:^ Difference between the patients and HC, *p*_*2*_^:^ Difference between subgroups of patients

^a^Median ± SD

^b^Median (IQR)

*Statistically significant

**Table 2:** Brain global and regional iron concentrations in the studied sample

	MS patients (*n*= 500 ROIs)				HC (*n*= 500 ROIs)	P_0_	P_1_	P_2_	P_**3**_
	Total	Benign	Aggressive	Naive					
**Brain volume in cm**^**3** a^	1,903	1,909	1,887	1,911	2,075	0.047*	0.924	0.995	0.789
**Global iron load in ppb, median**^b^	-4.2808	-4.1009	-4.6923	-4.1983	-2.9640	0.647	0.450	0.940	0.545
**Iron load ppb in cm**^**3**b^	-20.994	-0.7859	-32.011	-28.164	-14.577	0.660	0.227	0.247	0.661
**Regional iron load in ppb**^b^									
– Basal ganglia	0.011	0.009	0.013	0.009	0.008	0.442	0.042*	0.439	0.392
– Thalami	-0.002	-0.002	-0.003	-0.001	-0.001	0.550	0.552	0.552	0.989
– NAWM	-0.007	-0.005	-0.009	-0.007	-0.012	0.031*	0.291	0.414	0.787
– MS lesions	-0.029	-0.004	-0.003	-0.041	0.004	< 0.001*	0.225	0.037*	0.249
– Periventricular	-0.027	0.009	-0.010	-0.044	0.009	0.001*	0.301	0.004*	0.061
– Juxtacortical	-0.033	-0.063	-0.012	-0.043	0.013	0.017*	0.576	0.945	0.475
– Infratentorial	-0.059	-0.070	-0.097	-0.016	-0.035	0.419	0.440	0.465	0.083
P_4_	<0.001*	0.025*	0.001*	< 0.001*	<0.001*				
P_5_	0.070	0.147	0.032*	0.417	0.169				

*cm*^*3*^ Cubic centimeter, *HC* Healthy controls, *n* Number, *NAWM* Normal-appearing white matter, *P*_*0*_ Difference between patients and controls, *P*_*1*_ Difference between benign and aggressive MS, *P*_*2*_ Difference between benign and drug naïve patients, *p*_*3*_ Difference between aggressive and naïve patients, *p*_*4*_ Differences between iron load in all locations within the same group, *p*_*5*_ Differences between iron load between MS lesions (or corresponding locations in healthy controls) within the same group, *ppb* Parts per billion

^**a**^Mean

^b^median

*Statistically significant

**Fig. 5:**
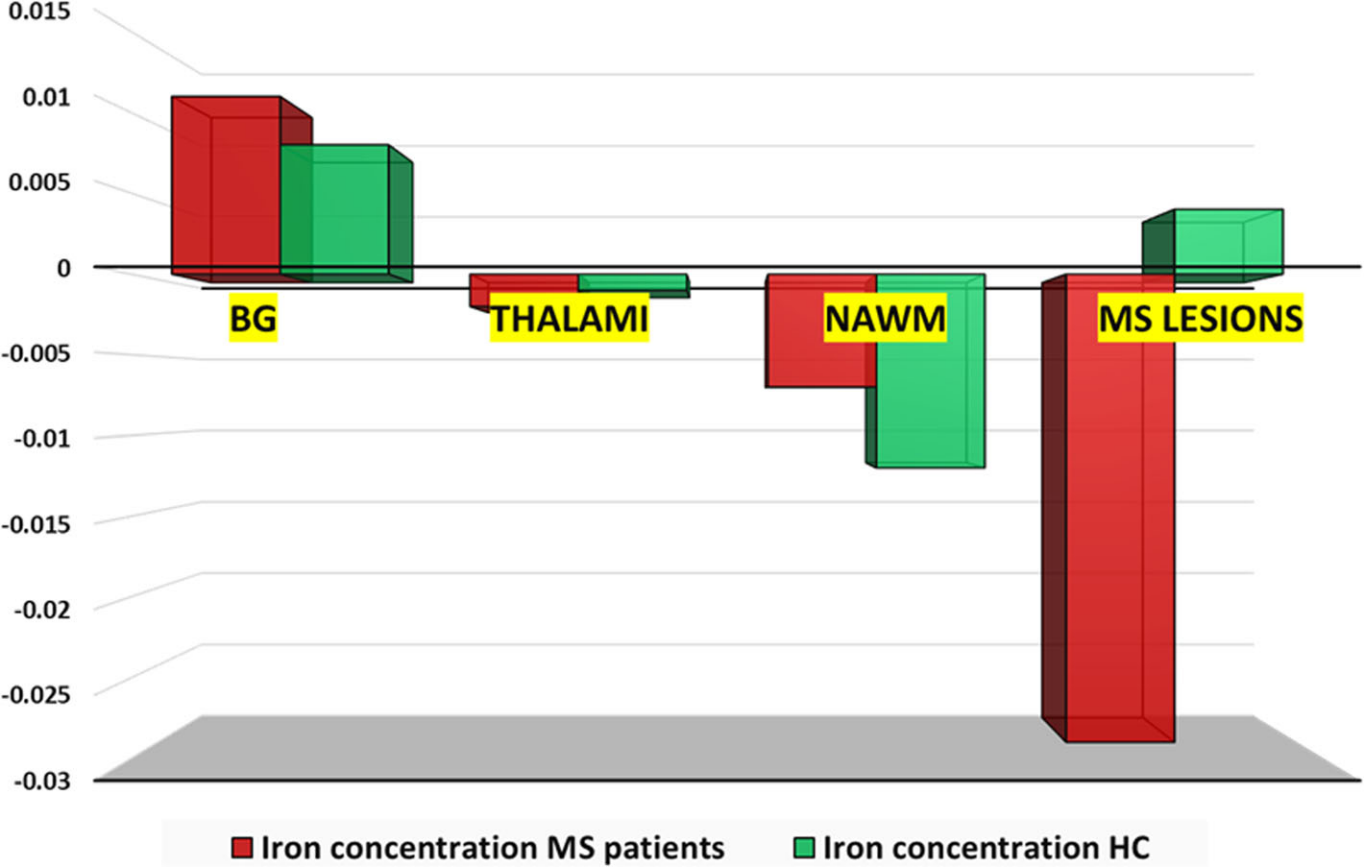
Regional iron concentrations in multiple sclerosis patients *versus* healthy controls

For the regional iron concentrations, patients had significantly high iron concentrations in NAWM (-0.007 ppb *versus* -0.012 ppb, *p* = 0.031) and significantly low iron concentrations in MS lesions (-0.029 ppb *versus* 0.004 ppb, *p* < 0.001), particularly the periventricular (-0.027 ppb *versus* -0.009 ppb, *p* = 0.001) and cortical/juxtacortical lesions (-0.033 ppb *versus* 0.013 ppb, *p* = 0.017) in comparison to the healthy controls (Fig. 5). The subgroup analysis showed significantly higher iron concentrations in the basal ganglia of aggressive MS than benign MS (0.013 ppb *versus* 0.009, *p* = 0.042) and significantly lower iron concentration in MS lesions in DMT-naïve patients than benign MS (-0.047 ppb *versus* -0.004, *p* = 0.037). No significant differences were seen in the regional iron concentrations in DMT-naïve and aggressive MS (*p* ≥ 0.061). Of interest, there was a significant difference between the iron concentrations between the different locations within each group (*i.e*., between the basal ganglia, thalami, NAWM, and MS lesions). Still, there was no difference in the iron concentrations between the MS lesion at different sites (*i.e.*, periventricular, juxtacortical, and infratentorial lesions). An exception was the aggressive MS subgroup, where iron concentrations were highest in the periventricular lesions (*p* = 0.032). It is to be noted that all MS lesions included in the analysis were chronic inactive lesions with no iron rim at their edges.

The correlation between the clinical profile and iron load (global and regional) is depicted in Table 3. Of note, no significant correlation was detected between the global iron load and any of the studied clinical parameters. The regional iron concentrations, in contrast, had a significant correlation with several parameters. Higher iron concentrations in the basal ganglia were correlated with worse EDSS scores (*p* = 0.029). Lower iron concentrations in the thalami were correlated with higher T25FW test scores (*p* = 0.027). Iron concentrations in the NAWM were inversely correlated with the disease duration (*p* = 0.012) and T25FW test (*p* = 0.039). Iron concentrations in the periventricular lesions were inversely correlated with the patients’ age (*p* = 0.023), disease duration (*p* < 0.001), EDSS (*p* = 0.019), and T25FW test (*p* = 0.001), and directly correlated with the interferon-beta duration (*p* = 0.021).

**Table 3:** Correlation between clinical profile and brain iron concentrations (global and regional) in MS patients

	Global iron load (Iron load per cc2 brain volume)	Regional iron load (*n* = 500 ROIs)			
		Basal ganglia	Thalami	NAWM	MS periventricular lesions
**Age**	*p* > 0.05	*p* > 0.05	*p* > 0.05	*p* > 0.05	*r* = – 0.163, *p* = 0.023*
**Disease duration**	*p* > 0.05	*p* > 0.05	*p* > 0.05	*r* = – 0.323, *p *= 0.012*	*r* = – 0.292, *p* < 0.001*
**Number of relapses**	*p* > 0.05	*p* > 0.05	*p* > 0.05	*p* > 0.05	*p* > 0.05
**INF-β duration**^**a**^	*p* > 0.05	*p* > 0.05	*p* >0.05	*p* > 0.05	*r* = 0.165, *p* = 0.021*
**EDSS**	*p* > 0.05	*r* = 0.200, *p* = 0.029*	*p* > 0.05	*p* > 0.05	*r* = – 0.167, *p* = 0.019*
**T25FW**	*p* > 0.05	*p* > 0.05	*r* = -0.285, *p* = 0.027*	*r* = – 0.268, *p* = 0.039*	*r* = – 0.240, *p* = 0.001*
**SDMT**	*p* > 0.05	*p* > 0.05	*p* > 0.05	*p* > 0.05	*p* > 0.05

*EDSS* Expanded disability status scale, *INF-β* Interferon beta therapy, *MS* Multiple sclerosis, *n* Number, *NAWM* Normal-appearing white matter, *r* Spearman coefficient, *ROIs* Regions of interest, *T25FW* Timed 25-foot walking test, *SDMT* Symbol digit modality test

^a^Evaluated in the patients who received treatment only

*Statistically significant

## Discussion

In this study, we aimed at assessing whether the iron deposition in the MS brain is a problem of quantity or distribution. The main findings were that the global iron load was not altered in MS compared to healthy controls, and the global iron concentrations did not significantly correlate with any of the clinical parameters. The iron concentrations in MS lesions and NAWM were altered considerably compared to controls, and the regional iron concentrations correlated with several clinical parameters, *i.e*., age, disease duration, EDSS, T25FW test, and DMTs duration.

In the previous literature, the vast majority of the studies focused on iron evaluation in the deep grey matter of MS patients, and a significant association between the iron load in these regions and the disability progression was reported [2, 9–12, 17, 18]. To the best of our knowledge, this is the first study to assess the global iron load in MS, not only the regional iron concentrations, and to study the correlation between the iron concentrations inside the MS lesions with the clinical profile.

The global iron load in our MS cohort was not significantly different from the global iron load in healthy controls even after adjustment for the loss of volume observed in MS patients. It seems that the noted aberrant iron deposition in MS is rather a problem of distribution, not overall global load. Additionally, no significant correlation was found between the global iron concentrations and any of the studied clinical parameters, which supports the idea that MS is not a disease of iron overload or deficiency. These findings are interesting as they might help explain why the previous therapeutic trials of chelation therapy and endovascular interventions failed to show a beneficial effect [31, 32]. Given these findings, the hypotheses that aberrant iron deposition in MS is due to altered iron influx or active clearance/elimination from the brain should be downweighed [6, 19]. Iron deposition in MS is likely a consequence of perturbed iron homeostasis, supported by the evidence of polymorphism of the genes encoding iron export from the cells, iron-binding, and iron transport in MS patients [43, 44].

In agreement with the previous literature findings, the regional iron concentrations in our MS cohort were different from the healthy controls. The methodology of measuring regional iron concentration in our study was different from what has been performed previously. We used fixed-size ROIs placed at selected areas rather than segmentation techniques. This allowed us to measure and compare the iron concentrations not only in the basal ganglia and thalami but also in the MS lesions and NAWM. In our cohort, the iron concentrations were high in the basal ganglia and low in thalami but not significantly different from the healthy control. The iron concentrations were significantly low in MS lesions and high in the NAWM compared to corresponding locations in healthy controls. High basal ganglia iron has been consistently reported in MS in the literature [37, 45–47].

In contrast, reports on thalamic iron concentrations were conflicting between the studies. Thalamic iron concentrations were reported to be lower [37], higher [48], or not significantly different from the healthy controls [49]. The iron load was reported to be reduced inside the MS lesions in histological studies [14]. It was reported to be shifted to the lesion periphery forming a rim in slowly expanding chronic active lesions [50, 51]. In our study, the iron load in NAWM was higher than the iron load in similar regions of healthy controls. In disagreement with this finding, Hametner et al. [9] reported low iron concentrations in their histopathological studies of the NAWM of four MS brains compared to three control brains. Similarly, their second study of formalin-fixed autopsies of 24 MS brains *versus* 18 controls revealed the same results [52]. Their findings, however, were exclusive to the NAWM around the MS lesions edges, which is not the case in our study. Similar low iron concentrations were reported in NAWM of MS patients in a cross-sectional radiological study using R2* sequence for iron quantification [53]. The patients recruited in their study had a longer disease duration (12.3 years *versus* 7.0 years in ours) [53]. This might explain their difference from our results, given the accumulating evidence that the iron overload in different brain regions, even the basal ganglia, is reduced over time [6, 33, 53].

Taken altogether, the aberrant iron deposition could be concluded to be due to a process of shifting iron from certain regions to others inside the brain without affection of the overall global brain iron load. In a recently proposed explanation, iron was proposed to be reduced in brain regions where progressive damage to iron-containing cells (*i.e.*, the oligodendrocytes and myelinated neurons) takes place, such as the MS lesions and thalami (the relay of several neurons where Wallerian degeneration is reflected) [19, 54] and increased in brain regions where chronic iron-rich microglia are activated such as the NAWM and basal ganglia [6, 14]. There seems to be a piece of evidence that the iron load is elevated in brain regions where chronic microglial activation and ongoing oligodendrocyte and myelin damage (the iron most rich structures in the brain) with subsequent iron deposition [6, 14, 55]. Over time, when the vast majority of myelin and oligodendrocytes are lost, the iron load is reduced [6, 19, 33]. This might explain the noted correlation between the regional iron concentrations and different clinical parameters in our cohort.

The patients’ disability (evaluated by the EDSS or T25FW test) was significantly correlated with high iron concentrations in the basal ganglia and lower concentrations in the thalami and MS lesions. Similarly, patients with aggressive MS had higher basal ganglia iron than benign patients reflecting more prominent pathology. This might also explain the differences in iron concentrations reported in the literature in different brain regions, as the concentrations largely depend on the disease duration [6, 19, 33, 49, 53]. In our cohort, MS patients on interferon-beta therapy had higher iron concentrations in MS lesions. The longer the interferon therapy was used, the less iron loss was observed in MS lesions. We propose that the interferon-beta therapy received might have reduced myelin damage and subsequent iron loss. However, there is no data in the literature that can substantiate such speculation; and the cross-sectional design of the study does not allow confirmation or negation of this explanation. Though we tried to adjust for the impact of disease duration on iron concentrations by selecting patients within a narrow range of disease duration (*i.e*., a range of 5 years), the duration was significantly associated with iron concentrations in the MS lesions in this relatively narrow range of years.

The main strength points of this study are that it is the first one assessing the global brain iron load in MS, to compare the iron concentrations inside the MS lesions as well as the deep grey matter and NAWM via fixed-sized ROIs to avoid brain volume issues and to measure the iron concentrations in corresponding locations to MS lesions in healthy controls.

However, this study has limitations. First, a selection bias could not be avoided to control for the confounding effect of several well-established clinical variables on iron concentration, such as the disease duration. Accordingly, the results should be cautiously interpreted and should not be generalised to patients outside the scope of the inclusion criteria. This makes the generalizability of the study results limited. Second, the ventricles and sulci could not be excluded during the measurement of global iron load. However, we do not expect the CSF iron to affect the global iron load measurement due to its dynamic nature. Finally, measurement of iron concentrations via fixed-sized ROIs in certain slices might be inaccurate if the iron concentrations were not evenly distributed inside the MS lesions, NAWM, or deep grey matter.

In conclusion, our results showed that the aberrant iron deposition in MS is likely a distribution problem rather than the overall iron load inside the brain. Iron global concentrations are comparable between MS patients and healthy controls, but the regional iron concentrations are significantly different with areas showing low iron concentrations (such as MS lesions and thalami) and others showing high MS concentrations (such as basal ganglia and NAWM).

## Supplementary information

**Additional file 1.**

## Abbreviations

3D: Three-dimensional
BBB: Blood-brain barrier
CNS: Central nervous system
DMT: Disease-modifying therapy
EDSS: Expanded disability status scale
FLAIR: Fluid-attenuation inversion recovery
Mango: Multi-image analysis graphical user interface software
MS: Multiple sclerosis
NAWM: Normal-appearing white matter
ppb: Parts per billion
QSM: Quantitative susceptibility mapping
ROI: Region of interest
SD: Standard deviation
SWI: Susceptibility-weighted imaging
T25FW: Timed-25-foot walking
