# Supplementary material for: Iron deposition in multiple sclerosis: overall load or distribution alteration?
Source: Eur Radiol Exp. 2022 Sep 8;6:49. doi: 10.1186/s41747-022-00279-9 (PMC9458829; doi:10.1186/s41747-022-00279-9)
Supplement: Supplementary file 1 — Additional file 1. [file 41747_2022_279_MOESM1_ESM.docx]

**Supplementary table 1:** Number of ROIs measured at different brain locations in recruited subjects

|  | **MS patients (n= 500)** | | | | **HC**  **(n= 500)** |
| --- | --- | --- | --- | --- | --- |
|  | **Total** | **Benign** | **Aggressive** | **Naive** |  |
| Basal ganglia  Thalami  NAWM  MS lesions   - Periventricular - Juxtacortical - Infratentorial | 120  60  60  260  192  43  25 | 40  20  20  79  57  15  7 | 40  20  20  97  72  15  10 | 40  20  20  84  63  13  8 | 120  60  60  260  192  43  25 |
| **HC:** Healthy controls, **MS:** Multiple sclerosis, **n:** number, **NAWM:** Normal-appearing white matter, **ROIs:** Regions of interest. | | | | | |
